# Supplementary material for: A Systems Hypothesis of Lipopolysaccharide-Induced Vitamin Transport Suppression and Metabolic Reprogramming in Autism Spectrum Disorders: An Open Call for Validation and Therapeutic Translation
Source: Metabolites. 2025 Jun 13;15(6):399. doi: 10.3390/metabo15060399 (PMC12195403; doi:10.3390/metabo15060399)
Supplement: Supplementary file 1 [file metabolites-15-00399-s001.zip › metabolites-3665973-supplementary/metabolites-3665973-Supplementary Table S2.pdf]

Transporter	Full Name	Functional Domain	Mechanism / Pathway Involved
SLC19A2	Thiamine transporter 1	Vitamin Transport	Thiamine uptake
SLC19A3	Thiamine transporter 2	Vitamin Transport	Thiamine uptake
SLC25A1	Citrate transporter (CIC)	Mitochondrial Transport	TCA cycle, lipid biosynthesis
SLC25A11	Mitochondrial 2-Oxoglutarate/Malate Carrier (OGC)	Mitochondrial Transport	Serine Mito Transport
SLC25A12	Aspartate-glutamate carrier 1 (AGC1)	Mitochondrial Transport	Malate-aspartate shuttle
SLC25A13	Mitochondrial transporting Glutamate and Aspartate	Mitochondrial Transport	Glu/Asp Mito Transport
SLC25A15	Mitochondrial basic amino acid transporter	Detoxification	Orn/Cit Mito Transport
SLC25A16	Transports coenzyme A (CoA)	Mitochondrial Transport	Fatty acid Metabolism
SLC25A29	Mitochondrial basic amino acid transporter	Mitochondrial Transport	Amino acid Metabolism
SLC25A32	Transports tetrahydrofolate (THF)	Mitochondrial Transport	One Carbon Metabolism
SLC25A38	Mitochondrial Glycine Transporter	Mitochondrial Transport	Amino acid Metabolism
SLC25A4	Adenine Nucleotide Translocator 1	Mitochondrial ATP/ADP exchange	Oxidative phosphorylation
SLC25A44	Mitochondrial BCAA Transporter	Mitochondrial Transport	BCAA metabolism
SLC2A1	Glucose transporter type 1	Glucose transport	Facilitated glucose uptake
SLC2A3	Glucose transporter type 3	Glucose transport	Facilitated glucose uptake
SLC46A1	Proton-coupled folate transporter	Vitamin Transport	Folate uptake (acidic pH)
SLC52A2	Riboflavin transporter 2	Vitamin Transport	Riboflavin Uptake
SLC5A6	Sodium-dependent multivitamin transporter	Vitamin Transport	Biotin, B5, Lipoate uptake

Supplementary Table S2.
Summary of cofactor-transporting genes identified in the PM³ model. For each transporter, the associated cofactors, metabolic domain, functional pathway, and subcellular localization are listed to support interpretation of transcriptomic vulnerability in ASD.
